# Copepods in Turbid Shallow Soda Lakes Accumulate Unexpected High Levels of Carotenoids

**DOI:** 10.1371/journal.pone.0043063

**Published:** 2012-08-16

**Authors:** Tobias Schneider, Alois Herzig, Karin A. Koinig, Ruben Sommaruga

**Affiliations:** 1 Laboratory of Aquatic Photobiology and Plankton Ecology, Institute of Ecology, University of Innsbruck, Innsbruck, Austria; 2 Biological Research Station Neusiedler See, Illmitz, Austria; 3 Institute of Ecology, University of Innsbruck, Innsbruck, Austria; US Dept. of Agriculture – Agricultural Research Service (USDA-ARS), United States of America

## Abstract

Carotenoids are protective pigments present in many aquatic organisms that reduce the photooxidative stress induced by short-wavelenght solar radiation, yet increase their susceptibility to predators. *Arctodiaptomus spinosus*, a calanoid copepod typically found in many fishless shallow soda lakes, shows large between-lake differences in pigmentation. Here, we attribute these differences to the environmental state of these ecosystems, namely, ‘dark water’ lakes with submersed vegetation and turbid ‘white’ lakes lacking macrophytes. Copepod carotenoid concentration in the turbid ‘white’ lakes was significantly (about 20-fold) higher than in the ‘dark water’ ones, although the latter systems were characterized by higher transparency. In addition, males had on a dry weight basis around three times higher carotenoid concentrations than females. Mycosporine-like amino acids (direct UV screening substances) were found in all cases, but in low concentration. The environmental conditions in these ecosystems were largely shaped by the presence/absence of submersed macrophytes Thus, in the turbid lakes, the strong wind-driven mixis allows for copepods to be brought to the surface and being exposed to solar radiation, whereas in ‘dark water’ ones, macrophytes reduce water turbulence and additionally provide shelter. Our results explain the counter-intuitive notion of strong red pigmentation in copepods from a turbid ecosystem and suggest that factors other than high UV transparency favor carotenoid accumulation in zooplankton.

## Introduction

Shallow lakes can be defined by polymixis and by their (theoretical) ability to sustain submersed macrophytes [1]. These widespread and productive aquatic systems often exhibit one of two distinct conditions, i.e., a ‘clear water’ state with a rich vegetation of macrophytes or a turbid one where phytoplankton dominates and light is strongly attenuated due to algal blooms and wind-induced resuspension of sediments [2].

Solar ultraviolet radiation (UVR) penetrating into aquatic systems places a threat on planktonic organisms either by directly altering the configuration of essential molecules (e.g., of DNA) or by generating reactive oxygen species (ROS), which in turn damage DNA and other cell components [3], [4]. While in oligotrophic lakes, the attenuation of UVR depends mainly on the concentration of dissolved organic carbon (DOC), in eutrophic and turbid waters, suspended particles are responsible for UVR attenuation too [5], [6]. To avoid UVR exposure, some zooplankton stay in a ‘depth refuge’ during the day, often accompanied by UVR-dependent vertical migration behavior on a diel basis (e.g., [7], [8]). The depth refuge available in an aquatic ecosystem can be estimated by the ratio of a lakes’s depth to which 1% of surface UVR penetrates to its maximum depth [9]. This relation is small when the actual depth refuge is large and vice versa. In habitats with deep UVR penetration relative to the maximum depth (i.e., with low depth refuge) or shallow lakes with important wind-driven mixis, zooplankton might not be able to avoid hazardous radiation levels, and thus rely on the accumulation of photoprotective compounds (PPCs) [4]. These substances either directly screen UV radiation, or reduce UVR-induced damage by quenching ROS [3]. Some PPCs are only synthetized by primary producers, but may be passed on to the heterotrophic host (e.g., in corals) or to consumers (e.g., copepods) [10]–[12].

Carotenoids, a large family of PPCs, are lipid-soluble pigments having in many cases strong antioxidant capabilities [10]. Hairston [13] found that secondary carotenoids increase survival rates of copepods exposed to blue wavelengths, the main absorbance region of carotenoids, however, in this study UVR was not considered as a potential threat. UV screening capabilities of carotenoids are rather weak, but they facilitate the quenching of ROS and so they indirectly act as photoprotective agents [14]. The expression of carotenoids can be induced by UV exposure [3]. Consequently, the highest carotenoid concentrations in copepods are usually found in shallow clear lakes located at high elevation (e.g., [9], [15]). Besides their role in photoprotection, Byron suggested that carotenoids have a stimulatory effect on copepods metabolism via temperature increase [15], [16]. This hypothesis was criticized by Hairston, who argued that the possible temperature gain is insignificant [17]. More recently, Byron’s hypothesis of pigment-mediated metabolic stimulation gained some support from observations of an inverse relationship between water temperature and carotenoid concentration in a Patagonian shallow lake copepod population [18]. Carotenoids make organisms look red, orange, or blue (when bound to proteins; [19]). Therefore, they may alter predation pressure by increasing their visibility or providing camouflage [20]. The influence of predation threat and its cross relations with UV threat on carotenoid accumulation in copepods has been assessed by several authors. In these studies, predators were either fish (e.g., [21]), or salamanders combined with damselfly larvae [22]. The higher the predation threat was in relation to UV intensity, the lower was the carotenoid concentration in copepods, and the other way round.

Another family of PPCs is the mycosporine-like amino acids (MAAs), which in contrast to carotenoids, are direct UVR screening compounds without color [3], [11]. In a variety of organisms, MAA concentration is linked to the intensity of UV exposure in many marine and freshwater locations worldwide [23]. Both carotenoids and MAAs are believed to be obtained by copepods from their diet [10], [12]. Carotenoids and MAAs have been identified as complementary PPCs (i.e., one is high when the other is low), allowing copepods to adjust their protective strategy to environmental traits such as predation pressure [24], [25]. Copepods would accumulate MAAs rather than carotenoids in the presence of visual predators to reduce predation pressure [24]. However, this interpretation may not be valid in all systems, as certain larval fish see in the UV and harvest more efficiently in the presence of UVR [26].

Carotenoid pigmentation of *Arctodiaptomus spinosus* (Daday [27]), a calanoid copepod found in soda lakes and shallow (<1 m depth) alkaline ponds in the Pannonian Basin, eastern Turkey, Armenia, and Iran [28], varies remarkably among ecosystems in close geographic proximity. We observed that while in some lakes the copepods are nearly transparent, in others they are intensely colored orange-red. The pigments responsible for the coloration are carotenoids, and counter-intuitively to a presumed photoprotective role of these pigments, the most intensely colored specimens are found in highly turbid environments, where it seems implausible that short-wavelength solar radiation poses a significant threat.

The typical habitats of *A. spinosus* can be grouped into ‘dark water’ (relatively clear) and ‘white’ (turbid) lakes [29]. The ‘dark water’ lakes have rich submersed vegetation and brownish waters due to dissolved humic substances, as well as they lack suspended colloids. By contrast, ‘white’ lakes have only poor vegetation, soft fine sediments, and are very turbid. The whitish hue of their water is caused by suspended clay particles, which stay in suspension due to electrostatic interactions with the major cation Na^+^ in an alkaline milieu [30]. In the Pannonian lakes the clear ‘dark water’ lakes are seen as transition states in the course of ecosystem shrinkage [30]. Thus, those lakes evolve reversely as compared to the ecosystems described by Scheffer [1] that typically switch from clear to phytoplankton-turbid.

The aim of the present study was to test to which extent the accumulation of the supposed PPCs by *A. spinosus* can be explained by differences in optical lake properties, depth refuge, and water temperature, both among lakes and on a seasonal scale. Thus, we investigated two lakes of each state, hypothesizing that (1) environmental conditions differ among ‘dark water’ and ‘white’ lakes (rather than among lakes in each group, or within each lake on a seasonal scale). Likewise, we tested whether (2) copepods in the two groups of lakes accumulate different concentrations of carotenoids, and to which extend (3) within-lake changes in copepod pigmentation reflect the seasonality of environmental parameters.

## Results

### Environmental Conditions

**Figure 1:**
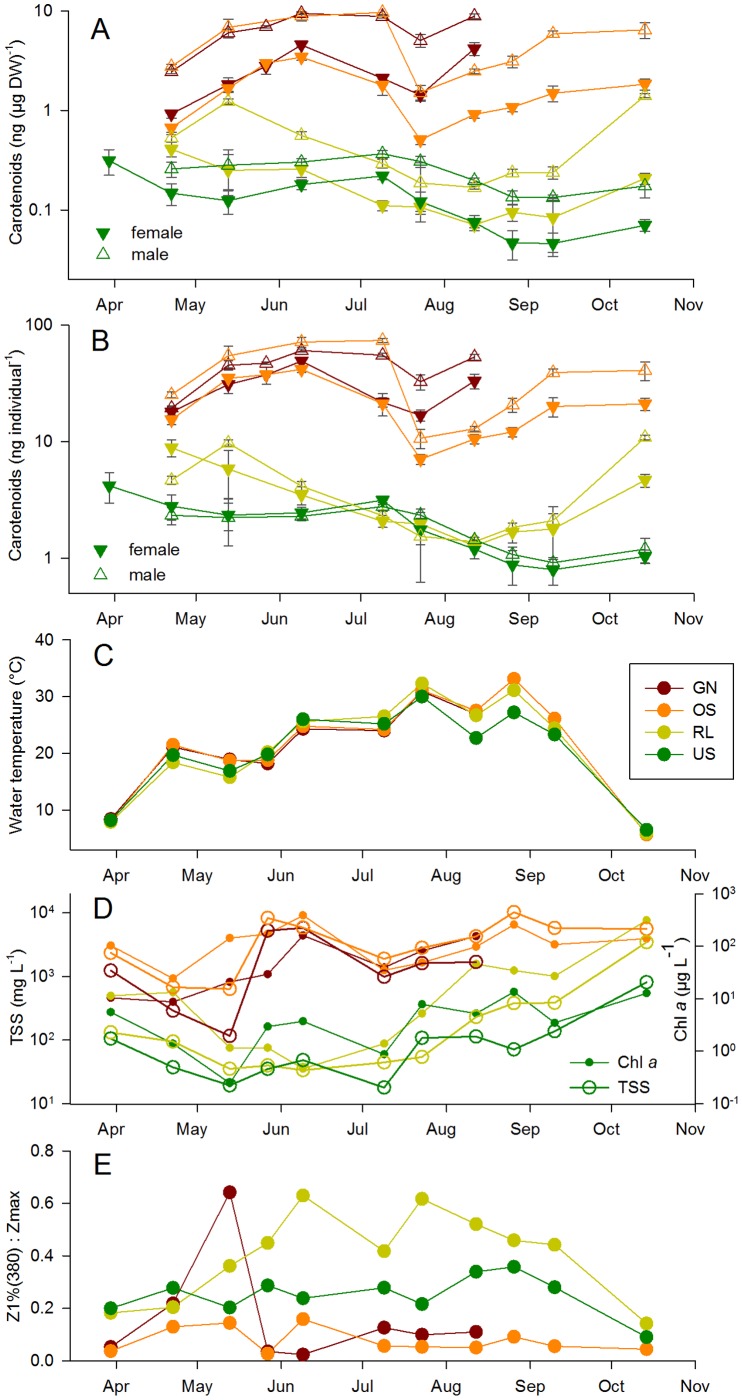
Seasonality of carotenoid concentrations in *A. spinosus* and environmental parameters. (A) carotenoid concentration normalized to dry weight, (B) carotenoids per individual copepod, (C) water temperature, (D) total suspended solids and chlorophyll *a*, and (E) Z_1%(380)_ : Z_max_ as an estimate of UV exposure (inverse of depth refuge). The four studied lakes are indicated by different colors. Carotenoids, TSS and Chl *a* are displayed in log scale. In A and B, full downward triangles represent females’ values, open upward triangles males’ values. In D, the solid line and symbols represent TSS, the dotted line Chl *a*.

Physicochemical and optical parameters in the four lakes underwent strong temporal changes during the study period (March to October). One of the turbid ‘white’ lakes, Lake GN, dried-out from 26 August onwards (Fig. 1). From 22 April and 13 May to the end of the study period, the bottom of the ‘dark-water’ Lake US was loosely covered by the macrophytes *Potamogeton pectinatus* and *Chara canescens*. The second ‘dark-water’ lake (RL) was dominated by floating aggregations of *C. canescens* from 22 April to 10 September. No submersed macrophytes were observed in the turbid ‘white’ lakes OS and GN.

**Figure 2:**
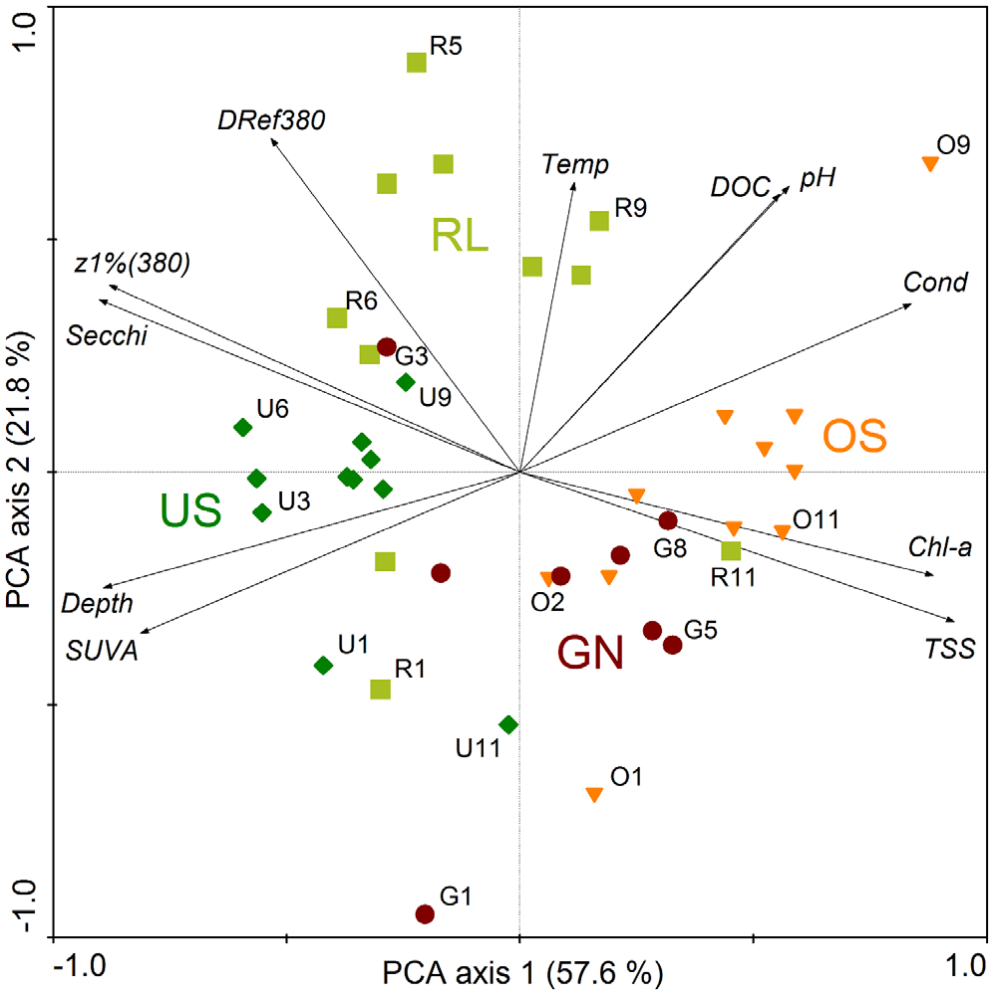
PCA biplot of samples (i.e., observations of one lake on one date) and environmental variables. The two groups of lakes (‘dark water’ lakes RL and US vs. ‘white’ lakes OS and GN) are significantly separated on a diagonal gradient (compare Table S3). Selected observations are labeled indicating lake (RL, US, OS, GN) and sampling date (1–11). Secchi depth, TSS and Chl *a* have been log-transformed. Light green squares, RL; dark green diamonds, US; orange triangles, OS; red circles, GN. Chl-*a* = chlorophyll *a*, Cond = conductivity, Depth = water level, DOC = dissolved organic carbon, SUVA = DOC-specific UV absorptivity at 254 nm, Temp = temperature, TSS = total suspended solids, z1%(380) = 1% attenuation depth at 380 nm, DRef380 = depth-related UV exposure (Z_1%(380)_ : Z_max_, inverse to the depth refuge at 380 nm).

**Figure 3:**
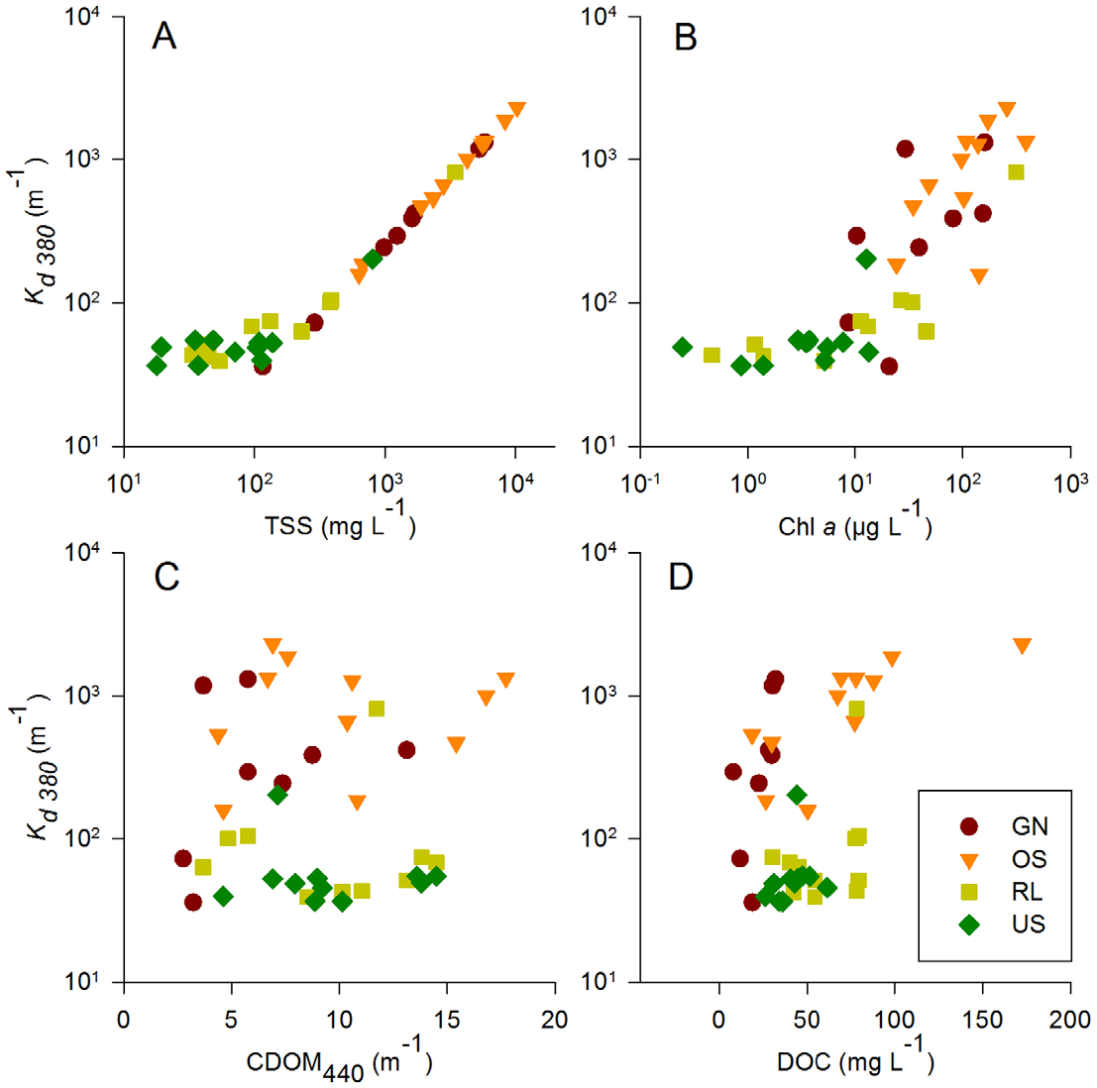
UV attenuation vs. environmental variables. Attenuation coefficient at 380 nm (*K_d_*_ 380_) plotted as a function of (A) total suspended solids, (B) chlorophyll *a*, (C) CDOM absorption at 440 nm, and (D) DOC concentration. The parameters in A–C were used to calculate the *K_d_* values (see methods).

The two groups of lakes were separated with some overlap along axes 1 and 2 of the principal component analysis (PCA) biplot (Fig. 2). According to the biplot, the ‘dark water’ lakes were characterized by higher water transparency (Secchi depth), deeper 1% UV-A penetration (380 nm), and lower depth refuge in the UV-A (i.e., a larger ratio of Z_1%(380)_ : Z_max._). The same was true for UV-B and PAR, but those are not shown in the PCA. By contrast, the turbid ‘white’ lakes showed high concentrations of chlorophyll *a* and total suspended solids (TSS), and, consequently, high UV and PAR attenuation (Fig. 2, Fig. 3A, B, Table S1). CDOM absorption and DOC concentration did not significantly differ between ‘dark water’ and ‘white’ lakes (ANOVAs; Fig. 3 C, D). In the ‘dark water’ water lakes, CDOM absorption contributed more to the UV-A attenuation coefficient (*K*_d_, on average 53% of the total *K*_d 380_ in RL and 63% in US) than TSS (46% and 37%, respectively). By contrast, the high attenuation coefficients in the ‘white’ lakes were due to the high partial attenuation by TSS (93% of *K*_d 380_ in OS, 89% in GN) (Fig. 3A). Although chlorophyll *a* concentration was correlated with *K*_d 380_ (R = 0.740, N = 41, P<0.001), it did have relatively little impact on the underwater radiation climate, as its contribution to attenuation was never more than 4% in any of the lakes (Fig. 3B). Overall, all *K*_d_ values were strongly correlated with the TSS content (R >0.99 for each wavelength *K*_d_ vs. TSS, N = 41, P<0.001) and thus, they were excluded in the PCA to avoid bias (i.e., only TSS was used). The PCA revealed that other parameters such as water temperature, DOC concentration, and pH did not differ between ‘dark’ and ‘white’ lakes, but rather among individual lakes within those groups. Conductivity, which is high in these systems (above 1800 µS cm^−1^), was inversely correlated with the water levels for the whole data set (R = –0.775, N = 41, P<0.001). The DOC-specific UV absorption (SUVA index) was positively correlated with the water level over all study sites (R = 0.791, N = 41, P<0.001). Water temperatures showed the expected seasonality, but did not differ among ‘dark water’ and ‘white’ lakes (ANOVA; Fig. 1C).

In spite of these similarities, the PERMANOVA analysis showed that for the whole assemblage of environmental variables, differences between the two groups, ‘dark water’ and ‘white’ lakes, were highly significant (*P*<0.01) and accounted for 36% of the total variation of environmental parameters (Table S2). Other highly significant factors were the date, and the lake within each group, explaining 23% and 18% of the total variation, whereas the weakly significant interaction factor of group and date accounted for only 7% (Table S2).

### Copepod Carotenoids and MAAs

*A. spinosus* occurred in all lakes during the whole sampling period. The estimated dry weight of individual female copepods was generally about twice that of male copepods. Carotenoid concentration in copepods was higher in the ‘white’ lakes than in the ‘dark water’ ones (Fig. 1A, B, Table S1). Additionally, carotenoid concentration on a dry weight basis was on average three times higher in male copepods than in females (Fig. 1A). However, this inter-sex difference in pigmentation was not significant (ANOVA) when carotenoids were expressed per individual copepod instead of per unit dry weight (Fig. 1B). PERMANOVA analysis revealed a highly significant difference in carotenoid concentration between the two groups (state) of lakes, as well as between female and male copepods (Table S3A, B). The lake state explained 73% of the carotenoid (on a dry weight basis) variation, while the animals’ sex and the interaction of lake state and sex explained additionally 12% and 4%, respectively (Table S3A). Significant differences between individual observations within each group, as indicated by the interactions of lake and date, and of lake, sex and date, accounted for 5% of the variation. Overall, temporal differences were close to significant (P = 0.055), but accounted for only 2% of the variation. No interactions between lake state and date, or between sex and date were observed. For carotenoids per individual, PERMANOVA revealed the same significant factors, but with a stronger influence of the lake state (82% explanation) relative to the inter-sex pigmentation difference (2%) (Table S3B).

Two MAAs were identified in copepods, namely shinorine and porphyra-334, but they were present in very low concentrations (Table S1). A PERMANOVA analysis on total MAAs revealed no significant effect of the lake state. The explained variation was distributed among the significant factors: sex, date, lake, and all their interactions accounting for 91% of the total variation.

### Influence of Environmental Parameters on Copepod Carotenoids and MAAs

When assessing the driving environmental factors of the variation in copepod-related parameters across lakes with a redundancy analysis (RDA), we detected a major influence of TSS on females’ and males’ carotenoid concentrations (49% and 47%, respectively). Additional significant explanatory parameters in both sexes were DOC (11%), and pH (6% females, 5% males) (Table S4).

To identify drivers of seasonal variation within lakes, the minimal set of significant explanatory variables for (a) female carotenoids and (b) male carotenoids was defined for each lake separately within the RDA. This resulted in different explanatory parameters for each lake, as well as for males and females. Thus, no environmental variable could be identified to generally predict within-lake variation of copepod carotenoids. RDA assessment of significant explanatory variables for MAAs failed to reveal consistent patterns, i.e., factors differed for males and females over all study sites, as well as for individual lakes.

**Figure 4:**
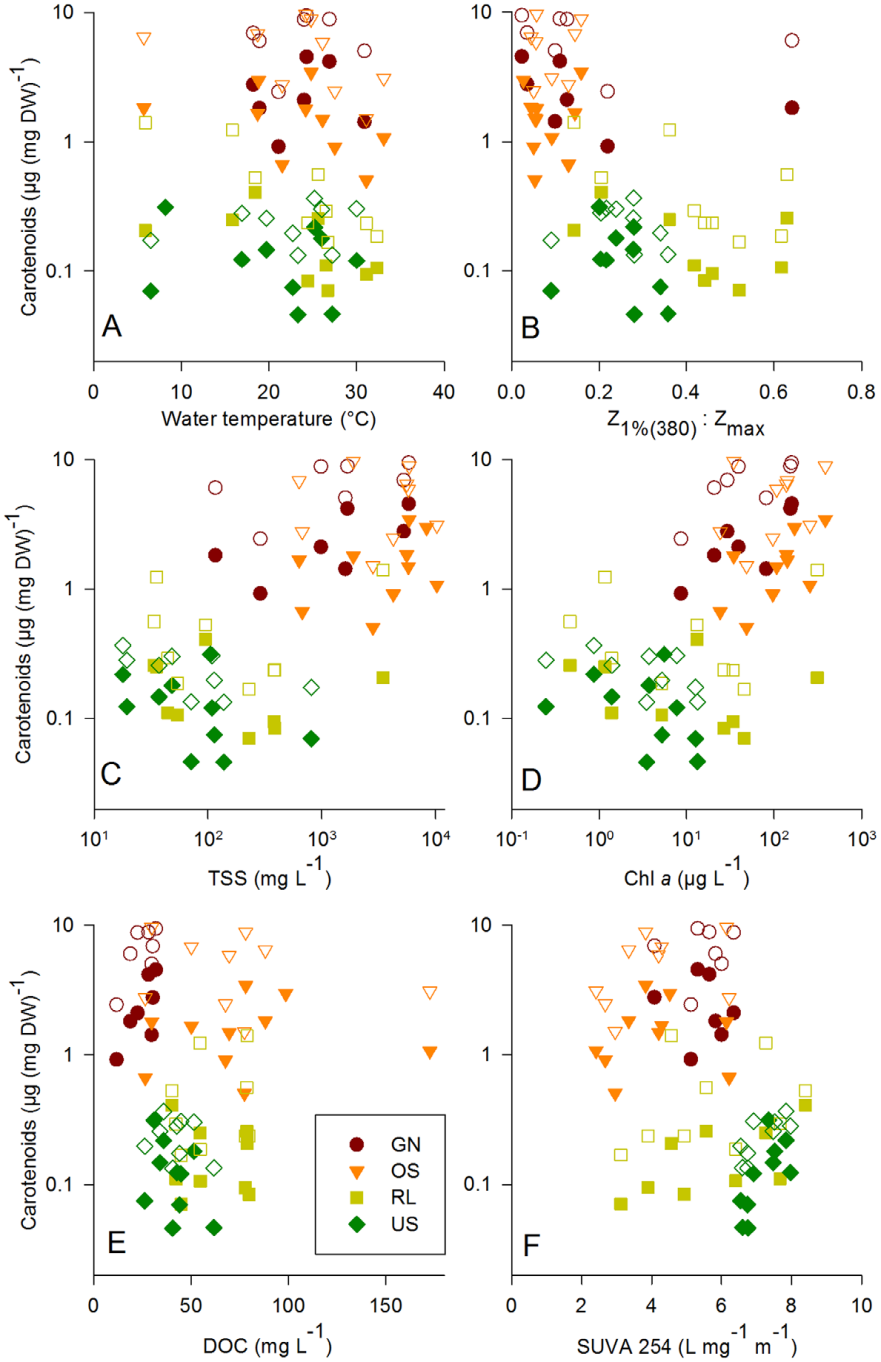
Copepod pigmentation related to environmental parameters. Carotenoid concentration in female and male *A. spinosus* plotted against (A) water temperature, (B) Z_1%(380)_ : Z_max_, (C) total suspended particles, (D) chlorophyll *a*, (E) DOC concentration, and (F) the SUVA index. Full symbols, females; open symbols, males.

In addition to the RDAs, we tested with linear regressions whether UV exposure conditions (expressed as Z_1%(380)_ : Z_max_) and water temperature explained copepod pigmentation. Neither of the two variables had a significant effect on carotenoid concentration in female or male copepods (Fig. 4A, B).

## Discussion

### Two States of Lakes

The four investigated lakes represent two distinct types of ecosystem state, i.e., the macrophyte-populated ‘dark water’ lakes (RL and US) and the extremely turbid ‘white’ lakes (OS and GN) (Fig. 1 D, E, Fig. 2). The two states of shallow lakes can be distinguished by their UV penetration/depth refuge, total suspended solids, and chlorophyll *a* concentration, as well as by the presence/absence of submersed vegetation. ‘Dark water’ and ‘white’ lakes are highly dynamic ecosystems and may change from year to year or even within a given year. For example, chlorophyll-*a* and total suspended solids concentrations for Lake US were much higher (up to 402 µg L^–1^ and 2980 mg L^–1^, respectively) in summer 2000 [31] than in the present study (Fig. 1 D, E, Table S1). This high variability was also observable in our study on 14 September, when submersed macrophytes disappeared in RL and total suspended solids and chlorophyll-*a* concentrations increased concomitantly by one order of magnitude (Fig. 1D, E). Despite this variability, we argue that the main driver of the differences between the two groups of lakes is the presence or absence of submersed and also emersed macrophytes which control the intensity of wind-induced mixis and, consequently, the concentration of suspended sediment particles in the water column. The difference in water transparency and depth refuge results as a consequence of the contrasting total suspended solids and chlorophyll-*a* concentrations in those ecosystems (Fig. 3, Fig. 4D).

### Photoprotective Compounds

The carotenoid concentrations in *A. spinosus* showed both, large between-lake and temporal variability. Copepods in the turbid ‘white’ lakes accumulated about 20 times as much pigments as those in the macrophyte-containing ‘dark water’ ones (Fig. 1A, B). These significant differences suggest that the state of a given shallow lake may not only affect abiotic parameters, but also phenotypic traits of zooplankton such as pigmentation. Interestingly, carotenoid concentrations accumulated by copepods in the turbid lakes were similar to those found in populations from very UV transparent high-latitude and high-altitude lakes rather than in lowland lakes (Table S5). This is surprising, as those systems are very different in regard to salinity, DOC concentration, UV exposure conditions, and phytoplankton biomass. By contrast, in copepod populations of the ‘dark water’ lakes US and RL, only low concentrations of carotenoids were found as compared to the literature (Table S1, Table S5). Copepods from those lakes were almost transparent, showing only a slightly red dot near to their mouth opening. It is important to emphasize the large seasonal variations in pigment content, especially in the intensely colored populations of the ‘white’ lakes (Fig. 1A, B). However, the difference in pigmentation between the two groups of lakes was at all times pronounced and highly significant.

Another interesting finding was that in all four lakes studied, male copepods accumulated more carotenoids per unit of dry weight than females, with an average ratio between 2.3 in US and 3.6 in OS (Fig. 1A). However, the difference was much lower or inexistent when carotenoids were expressed per individual copepod (Fig. 1B) and can thus be partly attributed to the different dry weight of males and females (the latter were larger and heavier). One possible reason for this inter-sex pigmentation difference is pigment translocation to eggs by females, presumably to provide photoprotection for the early larval stages [32]. Since in our study, eggs were removed from female *A. spinosus* prior to analysis, their pigment concentration was not quantified. Efforts to measure the carotenoid concentration in eggs were unsuccessful. However, the transfer of PPCs (in this case of MAAs) from females to nauplii in a cyclopoid copepod has been argued based on the high concentration of MAAs found in eggs and in early larval stages [33]. Considering that *A. spinosus* produces 4–5 generations per year as found, for example, in close-by Lake Neusiedl [34], the lower carotenoid concentrations in females may thus be a result of their investment in reproduction.

MAA concentrations in copepods were among the lowest compared with other studies (e.g. [9], [12], [24], [25]). In habitats with hazardous intensities of UV radiation, i.e., shallow UV clear lakes, MAAs are expected to be found in high concentrations [4], [35], [36]. Thus, we suggest that the low MAA concentrations found in our study result from the organisms not being exposed to high accumulated UV irradiance in these turbid lakes.

### Environmental Drivers of Copepod Pigmentation

As revealed by PERMANOVA analyses, 73% of the variation in carotenoid concentration (82% of carotenoids per individual) in *A. spinosus* was explained by the lake state (Table S3A, B). However, the two groups of lakes differed in several environmental properties (Fig. 2). Thus, our dataset does not allow for attributing differences in carotenoid pigmentation to a particular environmental factor. Nevertheless, we can advance probable mechanisms involved based on what we know about these ecosystems.

One well-documented function of carotenoid accumulation in copepods is photoprotection against the effect of solar radiation, particularly of wavelengths in the blue range [3], [22], [37]. Both UVR and PAR were on average stronger attenuated in the turbid ‘white’ lakes than in the ‘dark water’ ones. Further, the ratio of the water column to which 1% of UVR and PAR penetrates (Z_1%UV_ : Z_max_) as an inverse measure of ‘depth refuge’ was generally larger in the relatively deeper ‘dark water’ lakes (Fig. 1E). Nevertheless, copepods in the ‘white’ turbid lakes had more than one order of magnitude higher pigmentation than in the ‘dark water’ ones (Fig. 4B). This is in contrast to previous findings where the depth refuge had a high explanatory value of the among lake variability in carotenoids and MAAs concentrations in copepods of high UV environments (i.e., large Z_1%UV_ : Z_max_) [9], [35].

In shallow lakes with submersed vegetation, zooplankton may perform diel vertical and diel horizontal migration between macrophyte beds and the pelagic zone [38]. Copepods in the ‘dark’ lakes RL and US may benefit from the spatial heterogeneity and shadow generated by submersed macrophytes, as well as from the reduction in wind-induced turbulence caused by extensive *Phragmites* belts covering their northwestern shores (i.e., the prevailing wind direction [30]). Therefore, it is plausible to speculate that one reason why copepods in these ‘dark water’ lakes do not need photoprotection is that they are able to stay away of the surface or open water and avoid exposure to solar radiation.

In the ‘white’ lakes, the high turbidity values observed (i.e., high TSS concentration, Fig. 4C) and the strong wind, characteristic for the region [34], suggest that turbulence is a major force in these wind-exposed systems. The strong wind-driven mixis in these shallow systems could be a crucial factor that allow for frequent transport of the copepods to the surface (as they were visible as red dots on the grey water surface). As observed in other shallow turbulent lakes, if the mixing force is strong enough to prevent the animals from staying at a certain depth, this would lead to regularly repeated short-term sunlight exposure [39].

Carotenoid accumulation in copepods is limited by their availability in the diet, i.e., quality and quantity of edible phytoplankton from which these pigments are ultimately derived [10]. Indeed, in the less turbid ‘dark water’ lakes populated by transparent *A. spinosus*, much lower chlorophyll *a* concentrations were found than in the more productive ‘white’ lakes (Fig. 4D). However, chlorophyll *a* was correlated with other environmental factors such as conductivity, TSS, UV attenuation, or (inversely) with water level. Thus, from a relationship found between one of these factors and carotenoid concentration in copepods, we can hardly tell which one really is a potential driver. A similar situation where a correlation between chlorophyll *a* and carotenoids in copepods is argued to be accidental is explained by Hairston for two alkaline lakes in the Lower Grand Coulee, Washington, USA [22].

While population density was not assessed in the present study, according to our perception during sampling, *A. spinosus* was generally much more abundant in the ‘white’ lakes than in the ‘dark water’ ones. These observations are in agreement with direct measurements done on 9 June 2009, when abundance was 1654 ind L^−1^ in OS and 1296 ind L^−1^ in GN, compared with 315 ind L^−1^ in RL and 96 ind L^−1^ in US (personal communication by Z. Horváth, Department of Systematic Zoology and Ecology, Eötvös Loránd University, Budapest, Hungary). High population densities as observed in the turbid lakes can lead to increased oxidative stress in aquatic animals [40]. For example, copepods cultured at unnaturally high densities exhibit molecular stress responses that affect egg production, but can be mitigated by antioxidants [41]. Carotenoids may support immune defense in copepods [42] which might be especially important in crowded situations with additional physical stress due to suspended colloids [43].

A disadvantage of bright pigmentation is the increased susceptibility to visually-oriented predators [21], [22]. Copepods have been shown to adjust their pigment concentration in response to counter-acting threats from predation and hazardous radiation [21], [24]. While single fish specimen may be introduced by birds from the close-by Lake Neusiedl, no fish population is expected to survive a full season in any of the investigated lakes due to the strong changes in salinity and occasional dry-out. On the other hand, Odonata larvae have been found in two consecutive years in Lake US [44]. Damselfly nymphs and salamander larvae may affect copepod pigmentation [22] and selective pressure on colored individuals in the ‘dark water’ lakes could additionally explain the almost complete lack of copepod pigmentation found in these ecosystems.

In conclusion, the contrast in pigmentation of *A. spinosus* observed between lakes is driven by the state of the shallow lake, i.e., macrophyte-dominated versus inorganic turbid plus phytoplankton-rich. While the penetration of UV radiation alone fails to explain differences in copepod pigmentation among these lakes, several possible stressors such as wind-induced turbulence combined with short-term sunlight exposure and crowding are probably involved in explaining this pattern. In the macrophyte-dominated ‘dark water’ lakes, these stress factors are much reduced or absent. Overall, our results suggest that high UVR exposure is not the only factor that favors carotenoid accumulation in zooplankton.

## Materials and Methods

### Ethics Statement

The lakes investigated are part of the National Park Neusiedlersee-Seewinkel (Austria) and all necessary permits were obtained for the described field studies from the Biological Research Station Neusiedler See. Permission was given by the director of the Research Institute and co-author of this manuscript, Prof. Alois Herzig. The copepod species is not endangered or protected.

### Study Sites and Sampling

Four soda lakes were selected representing the two types of shallow ecosystems typical for this region [29]. These ecosystems are more properly categorized as salt pans, but for the sake of simplicity, we refer to them as lakes. Runde Lacke (RL) and Unterstinker (US) represent the relatively clear ‘dark water’ lakes, while Oberstinker (OS) and Große Neubruchlacke (GN) are extremely turbid ‘white’ lakes. All four sites are located just east of Lake Neusiedl within 5 km to each other (Table S6).

Samples were taken on 11 occasions from March 30 to October 14, 2009. Usually, sampling started at 1 PM with RL, followed by US and OS, and was finished at 5 PM with GN. This sequence was repeated at all sampling dates. Water samples were collected in a bucket using a long plastic scoop. This sampling method was used to avoid resuspension of sediments. Zooplankton was collected using a 250 µm–mesh plankton net (mouth diameter approx. 30 cm, length 1 m) and placed into a 2 L plastic container. This procedure was repeated several times, depending on the density of the copepods, which differed among lakes and sampling dates. Samples were kept at 4°C until further treatment.

### Environmental Parameters

Transparency was measured using a white Secchi disk (20 cm diameter) on a marked thread and water levels were measured with a marked rope with a weight (flat metal) in the center of the lake (regarded as maximum depth as the lakes have a flat bottom). In US and OS, we additionally recorded the existing gauge water levels. In cases where Secchi depth reached the ground, it was set equal to the water level. Temperature and conductivity were measured directly in the bucket using a WTW 315i conductivity meter equipped with a thermistor. Subsequently, subsamples were placed in precombusted (2 h, 500°C) glass bottles with glass stoppers (100 mL, Schott) for DOC analysis. Samples for measuring the concentration of total suspended solids and chlorophyll *a* were collected in 2 L plastic bottles.

For the analysis of CDOM absorption and DOC concentration, about 30 mL of lake water was filtered through a glass fiber filter (Whatman GF/F; precombusted for 2 h at 450°C, rinsed with 20 mL of Milli-Q water and 10 mL of lake water). Filtration was done by hand using an acid washed syringe with a stainless steel filter holder. To measure CDOM absorption, a portion of each filtrate was scanned between 250 and 750 nm at 1 nm intervals in either 1 cm or 2 cm-quartz glass cuvettes in a Hitachi U-2000 double-beam spectrophotometer referenced against Milli-Q water and corrected for the presence of particulate matter by the absorbance value at 750 nm. The CDOM absorption coefficient was calculated from the measured absorbance *D* at a chosen wavelength λ and the path length of the cuvette *r* (m) as *a*_λ_ (m^−1^) = 2.303 • *D*_λ_/*r*. To quantify DOC (as non purgeable organic carbon), the filtrates were acidified with 2 M HCl to pH 2 and kept at 4°C until they were analyzed with a Shimadzu TOC-VCPH analyzer. The DOC-specific UV absorption at 254 nm (SUVA, in L mg^−1^ m^−1^) was calculated by normalizing *a*_254_ to the DOC concentration.

Total suspended solids (TSS) were measured in a precision balance (nearest 0.01 mg ) after filtrating 5 mL to 300 mL of lake water onto pre-weighed GF/F glass fiber filters (previously washed with Milli-Q water) and drying the material at 55°C for a minimum of 24 h. Missing TSS values on 13 May in all lakes were fitted with a linear regression found between log-transformed data (TSS *vs.* Secchi depth) over all study sites (R^2^ = 0.95): log(TSS) = –1.5437 • log(Secchi) +3.7311.

In order to analyze phytoplanktonic chlorophyll–*a,* remains of macrophytes and large algal filaments were first removed by screening the water through a 250 µm net before being filtered onto GF/C glass fiber filters. Extraction in 90% alkaline acetone was facilitated by sonication of the filters (1 min, 40 W) on crushed ice. The cleared extracts were scanned between 400–750 nm in a Hitachi U-2000 double–beam spectrophotometer using a 5 cm glass cuvette against an acetone reference. Chlorophyll *a* was calculated following Lorenzen [45].

UVR and PAR attenuation coefficients (*K*_d_) were calculated from Chl *a* concentration, total suspended solids, and CDOM absorption (*a*_440_) following [6]:

*K*_d_  =  *a* + *b* • Chl *a* + *c* • TSS + *d* • (18.216 • *a*_440_–0.209)

with parameters *a*, *b*, *c*, and *d* for different UV wavelengths (305 nm, 313 nm, 320 nm, 340 nm, 380 nm and 395 nm) and PAR (400–700 nm) given in V.-Balogh et al. [6]. Because attenuation coefficients (*K*_d_) were indirectly derived, differences among lakes, as well as on the temporal scale are expected to be similar for different wavelengths. Indeed, all *K*_d_ values were strongly correlated among all observations. Thus, we used only one (*K*_d 380_) in the analyses as a representative wavelength. The partial attenuation coefficients of Chl *a*, TSS and *a*_440_ were calculated by multiplying each parameter with its respective coefficient *b*, *c* or *d* [6]. The 1%-penetration depth of solar radiation was calculated as Z_1%_ = 4.605/*K*_d_, and set in relation to the maximum depth (Z_1%UV_ : Z_max_) to estimate the depth refuge (note that this estimate is inverse to the actual depth refuge, i.e., a high ratio of Z_1%UV_ : Z_max_ indicates a reduced depth refuge).

### Copepod Sorting and Dry Weight

Part of the zooplankton sample was concentrated onto a small 250 µm mesh size net and poured into a large Petri dish with filtered lake water. The filtered lake water (GF/F, of same origin as the plankton sample) was used to avoid a potential osmotic shock, and to allow for zooplankton to empty their guts for 30 min at room temperature. Then, zooplankton was narcotized with CO_2_-enriched water and identified based on [27]. Twenty females and males of *A. spinosus* were sorted out under a stereo microscope by gripping one antenna with a pair of forceps and placing them into an Eppendorf vial. Care was taken that copepods were not damaged during this procedure. Six replicates (three for carotenoids, three for MAAs) were included for both females and males. From each lake, we measured the length of 20 female and 20 male individuals, and calculated the dry weight (DW) using the formulae given in Bottrell et al. [46] for this species. Copepodite stages, which dominated in early spring (30 March), were not included in the analysis.

### Carotenoids by Spectrophotometry

Copepods were extracted in 400 µL of 95% ethanol (v/v) and sonicated for 1 min (40 W) on ice. Vials were kept at 8°C for 12 h before storage at –80°C. For pigment quantification, the samples were centrifuged at 7000 g for 6 min; subsequently 350 µL of the supernatant was transferred into a quartz glass cuvette of 1 cm path length. Spectral absorbance was measured in a Hitachi double-beam spectrophotometer from 750 to 400 nm. Carotenoid concentrations were then calculated according to Hessen and Sørensen [47].

### MAAs by HPLC

Extraction was done in 400 µL of 25% aqueous methanol (v/v) and sonicated at the beginning of the extraction as described before. The samples were placed in a water bath at 45°C for 2 h and then stored at –80°C for further analysis using high performance liquid chromatography (HPCL). Prior to HPLC analysis, the samples were centrifuged at 16100 g for 30 min. Then, 100 µL of the supernatant was transferred into small vials of the HPLC autosampler, where they were kept at 15°C. Aliquots of 50 µL were injected into a Phenosphere 5–µm pore–size RP–8 column (4.6 mm inner diameter, 25 cm length, Phenomenex) protected by a Guard column (Security Guard, Phenomenex), for isocratic reverse phase HPLC analysis. The samples were run with a mobile phase of 0.1% acetic acid in 25% aqueous methanol (v/v) for 15 min at a flow of 0.75 mL min^−1^. Absorbance peaks were measured at 310, 320, 334, and 360 nm in a Dionex system equipped with a diode-array detector (scanning from 200 to 595 nm).

Individual peaks were identified by their relative retention time compared to parallel standards, and by their absorption spectra. The content of specific MAAs in each sample was calculated from the HPLC integrated area at the wavelength of maximum absorption [33].

### Statistical Analysis

Principal components analysis (PCA) and redundancy analysis (RDA) were done on centered and standardized data using CANOCO for Windows 4.5 [48]. The following data transformations were applied: log(Secchi +1), log(TSS), log(Chl *a*), and 4^th^ root (PPCs concentration) – the latter for both carotenoids and MAAs. Due to the strong weight of TSS in the calculation of *K_d_* values, the latter were excluded from the PCA to avoid bias. Within RDA, environmental variables were chosen manually by forward selection, and their significance was tested with 999 Monte Carlo permutations. Variables with probability value of P<0.05 were considered significant. We defined the set of significant explanatory variables for (a) female carotenoids, (b) male carotenoids, (c) female MAAs and (d) male MAAs. PERMANOVA (permutational MANOVA) analysis was applied on the same data as the PCA, as well as on 4^th^ root-transformed copepod carotenoid concentrations, using the software package PRIMER 6 & PERMANOVA+ [49] to test which factors explain the variability in environmental conditions and copepod pigmentation. For environmental variables, the factors tested were: group (i.e., ‘white’ vs. ‘dark water’ lakes), lake nested in group, date, and the interaction of group and date. For carotenoids, the analysis was performed on the replicates (three per sampling occasion), and the factors were: group, lake nested in group, date, sex (male vs. female), and all possible interactions. Effects of (a) lake state and temperature, and (b) lake state and Z_1%(380)_ : Z_max_ and possible interaction effects on carotenoid concentration were tested with linear regressions using the software JMP, Version 7 (SAS Institute Inc., Cary, NC, USA).

## Supporting Information

Table S1 **Mean value and range for selected environmental variables, as well as concentrations of carotenoids and MAAs in copepods from the four lakes.**
(DOCX)

Table S2 **Results from multivariate PERMANOVA analysis for differences in environmental conditions between ‘dark’ and ‘white’ groups of lakes, dates, and lakes within each group.** Environmental variables in the analysis were the same as in the PCA (Fig. 2). Secchi depth, TSS, and Chl *a* were log-transformed; and all data were centered and standardized prior to analysis.
(DOCX)

Table S3 **Results from univariate PERMANOVA analysis for differences in copepod carotenoid concentrations between ‘dark’ and ‘white’ groups of lakes, female and male copepods, dates, and lakes within each group.** Data was ^4^√ transformed, centered and standardized prior to analysis. A, carotenoid concentration normalized to dry weight; B, carotenoids per individual copepod. Bold values denote significant differences at P<0.05. Date and lake were treated as random effects. For abbreviations, see Table S2.
(DOCX)

Table S4 **Results of RDA showing significant explanatory variables for the variability in carotenoid concentrations in copepods for all lakes together.** Within the RDA, the minimal sets of statistically significant (P<0.05) explanatory environmental variables were determined for carotenoids in females and males.
(DOCX)

Table S5 **Carotenoid concentrations (µg [mg DW]^−1^) reported for different lakes and copepod species.**
(DOCX)

Table S6 **Geographical coordinates of the study sites.**
(DOCX)
