# Supplementary material for: Copepods in Turbid Shallow Soda Lakes Accumulate Unexpected High Levels of Carotenoids
Source: PLoS One. 2012 Aug 16;7(8):e43063. doi: 10.1371/journal.pone.0043063 (PMC3420862; doi:10.1371/journal.pone.0043063)
Supplement: Table S1 — Mean value and range for selected environmental variables, as well as concentrations of carotenoids and MAAs in copepods from the four lakes. (DOCX) [file pone.0043063.s001.docx]

**Table S1**. Mean value and range for selected environmental variables, as well as concentrations of carotenoids and MAAs in copepods from the four lakes.

|  |  | RL | | US | | OS | | GN | |
| --- | --- | --- | --- | --- | --- | --- | --- | --- | --- |
| Variable | Unit | Mean | Range | Mean | Range | Mean | Range | Mean | Range |
| Water level | cm | 19.3 | 4–34 | 36.3 | 25.3–47.3 | 10.9 | 2.2–23.2 | 17.8 | 10–30 |
| Secchi depth | cm | 13.7 | 1–26 | 18.8 | 2–42 | 0.5 | 0–3 | 2.9 | 0–11 |
| Temperature | °C | 21.3 | 5.9–32.3 | 20.5 | 6.5–30 | 21.8 | 5.7–33.1 | 21.6 | 8.4–30.9 |
| TSS | mg L^-1^ | 442 | 20.9–3449 | 191 | 17.9–807 | 4773 | 674–10248 | 2141 | 257–5808 |
| DOC | mg L^-1^ | 60.1 | 30.3–79.8 | 41.7 | 26.1–61.8 | 70.6 | 18.5–172.6 | 22.6 | 8–32 |
| Conductivity | mS cm^-1^ | 8.3 | 4.1–13.9 | 4.1 | 3.0–5.2 | 13.6 | 4.0–30.6 | 6.7 | 1.8–13.8 |
| Chlorophyll-*a* | µg L^-1^ | 41.2 | 0.5–312.4 | 5.2 | 0.2–13.4 | 137.2 | 24.3–385.3 | 63 | 8.7–159.6 |
| SUVA254 | L mg^-1^ m^-1^ | 6.0 | 3.1–8.7 | 7.3 | 6.6–8.2 | 4.2 | 2.4–6.2 | 6.1 | 4.1–10.2 |
| *K*_d_ _320 nm_ | m^-1^ | 117 | 85–1324 | 123 | 76–344 | 1651 | 261–3718 | 808 | 66–2121 |
| *K*_d_ _380 nm_ | m^-1^ | 63 | 39–811 | 61 | 37–202 | 1012 | 158–2304 | 493 | 36–1311 |
| *K*_d_ _PAR_ | m^-1^ | 13.3 | 6.5–324 | 15.6 | 4.8–77 | 410 | 61–950 | 198 | 11.8–539 |
| Z_1%_ _380_ | cm | 7.3 | 0.6–11.7 | 9.3 | 2.3–12.6 | 0.9 | 0.2–2.9 | 3.2 | 0.4–12.8 |
| Z_1% 380_ : Z_max_ |  | 44% | 14%–63% | 25% | 9%–36% | 8% | 3%–16% | 16% | 2%–64% |
| *f*-carotenoids | µg/(mg DW) | 0.2 | 0.1–0.4 | 0.1 | 0.1–0.2 | 1.6 | 0.5–3.4 | 2.5 | 0.9–4.5 |
| *m*-carotenoids | µg/(mg DW) | 0.5 | 0.2–1.4 | 0.2 | 0.1–0.4 | 5.3 | 1.5–9.6 | 6.8 | 2.4–9.4 |
| *f*-MAAs | ng/(mg DW) | 40.8 | 3.5–96.6 | 20.5 | 3.5–39.6 | 128.9 | 3.1–340.6 | 30.2 | 3.6–96.4 |
| *m*-MAAs | ng/(mg DW) | 189.5 | 10.8–277.9 | 54.7 | 9.5–108.1 | 168.6 | 9.6–489.5 | 54.1 | 10.6–108.3 |

TSS = total suspended solids; DOC = dissolved organic carbon; SUVA_254_ = DOC-specific UV absorption at 254 nm; *K*_d_ = diffuse attenuation coefficient; PAR = photosynthetically active radiation (400–700 nm); Z_1% 380_ = 1%-penetration depth at 380 nm; *f*- and *m*- = female and male *A. spinosus*.
