# Supplementary material for: Copepods in Turbid Shallow Soda Lakes Accumulate Unexpected High Levels of Carotenoids
Source: PLoS One. 2012 Aug 16;7(8):e43063. doi: 10.1371/journal.pone.0043063 (PMC3420862; doi:10.1371/journal.pone.0043063)
Supplement: Table S2 — Results from multivariate PERMANOVA analysis for differences in environmental conditions between ‘dark’ and ‘white’ groups of lakes, dates, and lakes within each group. Environmental variables in the analysis were the same as in the PCA (Fig. 2). Secchi depth, TSS, and Chl a were log-transformed; and all data were centered and standardized prior to analysis. (DOCX) [file pone.0043063.s002.docx]

**Table S2.** Results from multivariate PERMANOVA analysis for differences in environmental conditions between 'dark' and 'white' groups of lakes, dates, and lakes within each group. Environmental variables in the analysis were the same as in the PCA (Fig. 2). Secchi depth, TSS, and Chl *a* were log-transformed; and all data were centered and standardized prior to analysis.

| Source | df | P | VC |
| --- | --- | --- | --- |
| **Gr** | 1 | **0.004** | 36% |
| **Da** | 10 | **0.001** | 23% |
| **La(Gr)** | 2 | **0.001** | 18% |
| **Gr×Da** | 10 | **0.032** | 7% |
| Res | 17 |  | 16% |
| Total | 40 |  | 100% |

Bold values denote significant differences at P < 0.05. Date and lake were treated as random effects. VC: estimate of variance components, i.e., the relative contribution of each factor to total variation. Gr: group ('dark' vs. 'white'), Da: date, La(Gr): lake nested in group. “×” denotes an interaction effect.
