# Supplementary material for: Copepods in Turbid Shallow Soda Lakes Accumulate Unexpected High Levels of Carotenoids
Source: PLoS One. 2012 Aug 16;7(8):e43063. doi: 10.1371/journal.pone.0043063 (PMC3420862; doi:10.1371/journal.pone.0043063)
Supplement: Table S3 — Results from univariate PERMANOVA analysis for differences in copepod carotenoid concentrations between ‘dark’ and ‘white’ groups of lakes, female and male copepods, dates, and lakes within each group. Data was 4√ transformed, centered and standardized prior to analysis. A, carotenoid concentration normalized to dry weight; B, carotenoids per individual copepod. Bold values denote significant differences at P<0.05. Date and lake were treated as random effects. For abbreviations, see Table S2. (DOCX) [file pone.0043063.s003.docx]

**Table S3.** Results from univariate PERMANOVA analysis for differences in copepod carotenoid concentrations between 'dark' and 'white' groups of lakes, female and male copepods, dates, and lakes within each group. Data was ^4^√ transformed, centered and standardized prior to analysis. A, carotenoid concentration normalized to dry weight; B, carotenoids per individual copepod. Bold values denote significant differences at P < 0.05. Date and lake were treated as random effects. For abbreviations, see Table S2.

A.

| Source | df | P | VC |
| --- | --- | --- | --- |
| **Gr** | 1 | **0.001** | 73% |
| **Sex** | 1 | **0.001** | 12% |
| Da | 10 | 0.055 | 2% |
| La(Gr) | 2 | 0.082 | 1% |
| **Gr×Sex** | 1 | **0.001** | 4% |
| Gr×Da | 8 | 0.149 | 2% |
| Sex×Da | 9 | 0.129 | 0% |
| La(Gr)×Sex | 2 | 0.295 | 0% |
| **La(Gr)×Da** | 14 | **0.001** | 4% |
| Gr×Sex×Da | 8 | 0.367 | 0% |
| **La(Gr)×Sex×Da** | 13 | **0.001** | 1% |
| Residual | 135 |  | 1% |
| Total | 204 |  | 100% |
|  |  |  |  |

B.

| Source | | df | | P | VC |
| --- | --- | --- | --- | --- | --- |
| **Gr** | | 1 | | **0.001** | 82% |
| **Sex** | | 1 | | **0.007** | 2% |
| Da | | 10 | | 0.201 | 2% |
| La(Gr) | | 2 | | 0.127 | 1% |
| **Gr×Sex** | | 1 | | **0.030** | 2% |
| Gr×Da | | 8 | | 0.435 | 0% |
| Sex×Da | | 9 | | 0.173 | 0% |
| La(Gr)×Sex | | 2 | | 0.292 | 0% |
| **La(Gr)×Da** | | 14 | | **0.001** | 8% |
| Gr×Sex×Da | | 8 | | 0.147 | 1% |
| **La(Gr)×Sex×Da** | | 13 | | **0.001** | 2% |
| Residual | | 135 | |  | 1% |
| Total | | 204 |  | 100% |  |
