# Supplementary material for: Copepods in Turbid Shallow Soda Lakes Accumulate Unexpected High Levels of Carotenoids
Source: PLoS One. 2012 Aug 16;7(8):e43063. doi: 10.1371/journal.pone.0043063 (PMC3420862; doi:10.1371/journal.pone.0043063)
Supplement: Table S4 — Results of RDA showing significant explanatory variables for the variability in carotenoid concentrations in copepods for all lakes together. Within the RDA, the minimal sets of statistically significant (P<0.05) explanatory environmental variables were determined for carotenoids in females and males. (DOCX) [file pone.0043063.s004.docx]

**Table S4.** Results of RDA showing significant explanatory variables for the variability in carotenoid concentrations in copepods for all lakes together. Within the RDA, the minimal sets of statistically significant (P < 0.05) explanatory environmental variables were determined for carotenoids in females and males.

| females | | |  | males | | |
| --- | --- | --- | --- | --- | --- | --- |
| Variable | P | % expl |  | Variable | P | % expl |
| log TSS | 0.001 | 49.2 |  | log TSS | 0.001 | 47.0 |
| DOC | 0.008 | 10.5 |  | DOC | 0.008 | 10.8 |
| pH | 0.022 | 6.4 |  | pH | 0.049 | 5.0 |

% expl. = percentage of explained variation. For other abbreviations see Table S1.
