# Supplementary material for: Copepods in Turbid Shallow Soda Lakes Accumulate Unexpected High Levels of Carotenoids
Source: PLoS One. 2012 Aug 16;7(8):e43063. doi: 10.1371/journal.pone.0043063 (PMC3420862; doi:10.1371/journal.pone.0043063)
Supplement: Table S5 — Carotenoid concentrations (µg [mg DW]−1) reported for different lakes and copepod species. (DOCX) [file pone.0043063.s005.docx]

**Table S5.** Carotenoid concentrations (µg [mg DW]^-1^) reported for different lakes and copepod species.

| Location | Species | Carotenoid concentration | Comments | Reference |
| --- | --- | --- | --- | --- |
| Two mountain lakes, Washington State | *Diaptomus kenai* | 2.3–7.49 |  | [50] |
| Alpine lake, Chile | *Boeckella gracilipes* | 2.3 |  | [51] |
| Alpine lake, Austria | *Cyclops abyssorum tatricus* | 6.5 |  | [51] |
| Transparent lake, Pennsylvania | *Leptodiaptomus minutus* | 0.4–4.5 | Minimum in September, maximum in April | [12] |
| High-altitude lake, Colorado | *Hesperodiaptomus shoshone* | 8.5 |  | [25] |
| Small lake in southern Sweden | *Eudiaptomus gracilis* | 4.49 |  | [37] |
| Coastal lake, Bering Strait | *Leptodiaptomus angustilobus* | 7.29 |  | [37] |
| Shallow lake in Patagonia | *Boeckella antiqua* | 1.4–3.5 | Minimum in October, maximum in August | [18] |
| 37 subarctic, temperate, and dry-temperate lakes | *Eudiaptomus* sp., *Leptodiaptomus* sp. | 0.8–2.6 | Minimum in dry-temperate, maximum in temperate lakes | [24] |
| Five high-altitude Himalayan lakes | *Arctodiaptomus jurisowitchi* | 1.0–17.0 | Free astaxanthin; correlated with depth refuge | [9] |
| Seewinkel, 'dark water' lakes | *Arctodiaptomus spinosus* | 0.0–1.4 |  | This study |
| Seewinkel, 'white' turbid lakes | *A. spinosus* | 0.5–9.6 |  | This study |

50. Hairston NG Jr (1978) Carotenoid photoprotection in *Diaptomus kenai*. Verh Int Ver Theor Angew Limnol 20: 2541–2545.

51.Tartarotti B (1999) Survivorship of *Cyclops abyssorum tatricus* (Cyclopoida, Copepoda) and *Boeckella gracilipes* (Calanoida, Copepoda) under ambient levels of solar UVB radiation in two high-mountain lakes. J Plankton Res 21: 549–560
