# Supplementary material for: Copepods in Turbid Shallow Soda Lakes Accumulate Unexpected High Levels of Carotenoids
Source: PLoS One. 2012 Aug 16;7(8):e43063. doi: 10.1371/journal.pone.0043063 (PMC3420862; doi:10.1371/journal.pone.0043063)
Supplement: Table S6 — Geographical coordinates of the study sites. (DOCX) [file pone.0043063.s006.docx]

**Table S6**. Geographical coordinates of the study sites.

| Lake | Abbreviation | Latitude (N) | Longitude (E) |
| --- | --- | --- | --- |
| Runde Lacke | RL | 47°47' | 16°48' |
| Unterer Stinkersee | US | 47°48' | 16°47' |
| Oberer Stinkersee | OS | 47°49' | 16°48' |
| Große Neubruchlacke | GN | 47°47' | 16°51' |
